# Epidemiology of rhinovirus under the COVID‐19 pandemic in Guangzhou, China, 2020

**DOI:** 10.1002/iid3.632

**Published:** 2022-05-11

**Authors:** Jianyun Lu, Tiantian Wu, Qing Zeng, Yiyun Chen, Yanhui Liu, Di Wu

**Affiliations:** ^1^ Director Guangzhou Baiyun District Center for Disease Control and Prevention Guangzhou Guangdong P. R. China; ^2^ Institute of Human Virology | Zhongshan School of Medicine | Key Laboratory of Tropical Disease Control of Ministry of Education Sun Yat‐sen University Guangzhou P. R. China; ^3^ Department of Biostatistics and Cancer Registration Guangzhou Center for Disease Control and Prevention Guangzhou P. R. China

**Keywords:** coronavirus disease 2019, human rhinovirus, outbreaks, pandemic, severe acute respiratory syndrome coronavirus 2

## Abstract

**Background:**

To analyze the epidemic characteristics of the human rhinovirus (HRV) outbreaks in Guangzhou, China, in 2020.

**Methods:**

Descriptive epidemiological methods were used to analyze the HRV‐related outbreaks in Guangzhou, 2020.

**Results:**

Seventeen outbreaks were reported in 2020 during the coronavirus disease 2019 (COVID‐19) pandemic in Guangzhou, a total of 465 patients (290 males and 175 females) were enrolled, with a median age of 10. A total of 223 (47.96%) had been tested for HRV, 89 (39.91%) of which were positive; 344/465 (73.98%) had a fever, 138/465 (29.68%) had a runny nose, 139/465 (29.89%) had a sore throat, 86/465 (18.49%) had a cough, 41/465 (8.82%) had a headache, and 37/465 (7.96%) had a sneeze. Patients at age of 13–15 had the highest rate of sore throat and runny nose, patients aged 11–12 had the highest rate of sneezing, and patients at age of 12–14 had the highest rate of positive rate. Patients tested positive had a higher rate of fever (*χ^2^* = 11.271, *p* = .001), cough (*χ^2^* = 6.987, *p* = .008), runny nose (*χ^2^* = 7.980, *p* = .005), and sneeze (*χ^2^* = 4.676, *p* = .031).

**Conclusion:**

The HRV was restored during the fighting of the COVID‐19 pandemic. The conventional COVID‐19 control measures were not effective enough in preventing rhinovirus. More appropriate control measures should be used to control HRV.

## 1 BACKGROUND

Human rhinoviruses (HRV) are the most common pathogens of the “common cold,” and caused more than half of the “common cold” was caused by HRV.^1^ HRV was designated as A, B, and C groups, within the genus *Enterovirus* and the family *Picornaviridae*.^2^ The main symptoms of the “cold” caused by HRV infection are low fever, cough, runny nose, and so forth, and was a self‐limited disease in most conditions.^3^ The coronavirus disease 2019 (COVID‐19) pandemic has led to an unprecedented level of concern about fever cases. The most common strategy for the control of the unexplained fever event during the COVID‐19 pandemic was to test for the nucleic acid of the severe acute respiratory syndrome coronavirus 2 (SARS‐COV‐2) and influenza virus (IFV); however, we detected and found both SARS‐COV‐2 and influenza were negative and proved to be HRV infection finally. Studies have reported that interference among respiratory viruses could affect the infection of the host at a large scale population level,^4^, ^5^, ^6^ IFV was the most observed and studied respiratory virus, Wu et al.^7^ found that rhinovirus disrupted the 2019 influenza A virus (IAV) pandemic in Europe and indicated that the respiratory viral interference can potentially affect the seasonal influenza epidemics and ongoing COVID‐19 pandemic. Piret and Boivin^8^ reported that HRV could reduce the likelihood of coinfection with IAV and reduce SARS‐COV‐2 replication in human airway epithelial cells. We analyzed the outbreaks of the HRV infection in Guangzhou, China, during the fight against the COVID‐19 pandemic, enhanced the understanding of the prevention and control of rhinovirus outbreaks, and thus provide better evidence for the interference of the respiratory virus during the COVID‐19 pandemic, and thus control the pandemic.

## 2 METHODS

### 2.1 Data and specimen collection

We collected all the information on the fever events of unknown cause during the combatting of the COVID‐19 pandemic in Guangzhou, 2020. Throat swabs or nasopharyngeal swabs were taken during all outbreaks of the fever events during the field investigation and were tested in the laboratory of the Guangzhou Center for Disease Control and Prevention.

### 2.2 Case definition

The cases enrolled in the study are the ones with one of these symptoms since the first case and before the last case was reported: fever, cough, runny nose, sneezing, or sore throat. If there were more than five cases reported in 3 days in a class should be defined as an outbreak.

### 2.3 Etiological detection

During the field investigation, throat swabs were collected from patients who did not take antiviral drugs within 3 days of onset and met the case definition. SARS‐COV‐2 and influenza nucleic acid were tested by real‐time quantitative polymerase chain reaction within 6 h after the samples were collected, and NxTAG Respiratory Pathogen Panel (LOT: XK051C‐1045; Luminex) was used to test for the respiratory polypathogen nucleic acid. IFV (subtype AH1, AH3), A (H1N1)pdm09, influenza B virus, respiratory syncytial virus (RSV), parainfluenza virus (PIV; 1, 2, 3, 4), bocavirus, human metapneumovirus (HMPV), HRV, adenovirus (ADV), coronavirus (OC43, 229E, HKU‐1, NL63), legionella, mycoplasma pneumonia, and chlamydia pneumonia were included. All tests were followed by the manufacturers' instructions.

### 2.4 Statistical analysis

Excel 2019 was used to collect the basic information about the patients in the outbreaks during the field investigation. *χ* ^2 ^test was used for statistical analysis using SPSS statistics (version 13.0), *p* < .05 was considered to be significant.

## 3 RESULTS

### 3.1 General information

Guangzhou is the capital city of Guangdong Province, China, with an 18.6 million population. We have detected 17 outbreaks of HRV in 2020, a total of 465 cases have been reported, of which, 290 were male and 175 were female, with an average age of 11.9 and the median age was 10.

### 3.2 Temporal and spatial epidemiology

Of all the 17 outbreaks, six were reported in June, three were reported in July, five were reported in September, one was reported in October, and two were reported in November. All outbreaks were reported in schools, 8 out of 17 were reported in primary school, six were reported in vocational education school, two were in kindergarten, and one in middle school (Table 1).

**TABLE 1:** Temporal and spatial epidemiology of the HRV outbreaks in Guangzhou.

School	School type	Time	No. of cases
School 1	Primary school	June	14
School 2	Middle school	June	16
School 3	Vocational education school	June	16
School 4	Primary school	June	21
School 5	Primary school	June	40
School 6	Primary school	June	55
School 7	Vocational education school	July	9
School 8	Vocational education school	July	29
School 9	Primary school	July	28
School 10	Vocational education school	September	87
School 11	Kindergarten	September	6
School 12	Kindergarten	September	60
School 13	Primary school	September	34
School 14	Primary school	September	9
School 15	Vocational education school	October	23
School 16	Primary school	November	5
School 17	Vocational education school	November	13

Abbreviation: HRV, human rhinovirus.

### 3.3 Etiological detection

Of all the 465 cases, 223 (47.96%) had been taken specimens and tested, all negative for SARS‐COV‐2 and influenza, of which, 89 (39.91%) were tested positive for HRV without genotyping.

### 3.4 Clinical characteristics of the patients in the outbreaks

We collected all the core health indicators of the enrolled patients, such as age, sex, the temperature of the patients, and the symptoms of the patients: cough, sore throat, muscle pain, headache, conjunctival congestion, runny nose, sneezing, diarrhea, and vomit.

We analyzed and found the average age of all the patients is 11.9 ± 5.9 years, and the median age is 10. A total of 290 males and 175 females were enrolled, 344/465 (73.98%) had a record of temperature over 37.3°C, 138/465 (29.68%) had a runny nose, 139/465 (29.89%) had a sore throat, 86/465 (18.49%) had cough, 41/465 (8.82%) had a headache, 37/465 (7.96%) had sneezed, and we found the female patients had a higher rate of having fever and more likely to be tested positive for the HRV test (Table 2). Notably, 7/465 (1.51%) had muscle pain, 5/465 (1.08%) had diarrhea, 1/465 (0.22%) had conjunctival congestion, and 1/465 (0.22%) had vomit.

**TABLE 2:** Sex‐specific clinical characteristics of the patients.

Characteristics	Sex		Total *N* (%)^a^
	Male *N* (%)^a^	Female *N *(%)^a^	
Total	290 (100)	175 (100)	465 (100)
Fever	200 (68.97)	144 (82.29)	344 (73.98)
Cough	56 (19.31)	30 (17.14)	86 (18.49)
Sore throat	85 (29.31)	54 (30.86)	139 (29.89)
Runny nose	84 (28.97)	54 (30.86)	138 (29.68)
Sneeze	23 (7.93)	14 (8.00)	37 (7.96)
HRV test			
Without test	146	96	242
Negative	91	43	134
Positive	53 (36.81)^b^	36 (45.57)^b^	89 (39.91)^b^

Abbreviation: HRV, human rhinovirus.

^a^ The percentage was the rate of the patients with the symptom in total of males or females.

^b^ The percentage was the positive rate of the tested males and females.

We next analyzed the results by age group and found the patients less than 5 years old had the highest rate of having fever, and if the patients had an elder age will have less rate of having a fever, but the patients over 18 had the opposite trend. The patients aged 9–10 had the highest rate of cough. Patients at the age of 13–15 had the highest rate of sore throat and runny nose, patients at the age of 11–12 had the highest rate of sneezing, and patients at age of 12–14 had the highest rate of positive rate (Table 3).

**TABLE 3:** Age‐specific clinical characteristics of the patients.

Age	Total cases *N*	Fever *N* (%)^a^	Cough *N* (%)	Sore throat *N* (%)	Runny nose *N* (%)	Sneeze *N* (%)	HRV test		
							Without test *N*	Negative *N*	Positive *N* (%)^b^
<5	54	49 (90.74)	4 (7.41)	4 (7.41)	0 (0)	0 (0)	36	12	6 (33.33)
<8	82	62 (75.61)	7 (8.54)	20 (24.39)	28 (34.15)	5 (6.1)	50	23	9 (28.13)
<10	79	56 (70.89)	28 (35.44)	32 (40.51)	35 (44.3)	13 (16.56)	30	26	23 (46.94)
<12	47	32 (68.09)	6 (12.77)	21 (44.68)	20 (42.55)	9 (19.15)	17	19	11 (36.67)
<15	25	15 (60.00)	7 (28.00)	13 (52.00)	13 (52.00)	4 (16.00)	12	2	11 (84.62)
<18	42	21 (50.00)	16 (38.1)	12 (28.57)	18 (42.86)	3 (7.14)	14	15	13 (46.43)
<20	72	53 (73.61)	10 (13.89)	16 (22.22)	14 (19.44)	0 (0)	35	22	15 (40.54)
≥ 20	64	56 (87.5)	8 (12.5)	21 (32.81)	10 (15.36)	3 (4.69)	48	15	1 (6.25)
Total	465	344 (73.98)	86 (18.49)	139 (29.89)	138 (29.68)	37 (7.96)	242	134	89 (39.91)

Abbreviation: HRV, human rhinovirus.

^a^ The patients with a body temperature ≥37.3°C are defined as having fever.

^b^ The percentage was the positive rate of the tested patients.

We next found that the tested patients had a statistically lower rate of fever than that of the patients without test (*χ* ^2^ = 32.563, *p* < .001), which might be due to our preference to take specimens of the cases with symptoms such as fever during field investigations. The rest of the clinical characteristics were no statistical difference between the tested and not tested ones (Table 4).

**TABLE 4:** The clinical characteristics of the patients in the outbreaks.

	HRV tested	Without HRV tested	***χ* ^2^**	*P*
Age				
Average	11.94	11.86	–	–
Sex				
Male	144 (64.6)	146 (60.3)	0.890	.345
Female	79 (35.4)	96 (39.7)		
Fever				
No	128 (38.1)	36 (14.9)	**32.563**	**<.001**
Yes	95 (61.9)	206 (85.1)		
Cough				
No	188 (84.3)	191 (78.9)	2.228	.136
Yes	35 (15.7)	51 (21.1)		
Sore throat				
No	165 (74.0)	161 (66.5)	3.084	.079
Yes	58 (26.0)	81 (33.5)		
Muscle pain				
No	222 (99.6)	236 (97.5)	3.228	.072
Yes	1 (0.4)	6 (2.5)		
Headache				
No	205 (91.9)	219 (90.5)	0.296	.586
Yes	18 (8.1)	23 (9.5)		
Conjunctival congestion				
No	223 (100.0)	241 (99.6)	0.923	.337
Yes	0 (0)	1 (0.4)		
Runny nose				
No	161 (72.2)	166 (68.6)	0.722	.396
Yes	62 (27.8)	76 (31.4)		
Sneeze				
No	202 (90.6)	226 (93.4)	1.247	.264
Yes	21 (9.4)	16 (6.6)		
Diarrhea				
No	221 (99.1)	239 (98.8)	0.128	.720
Yes	2 (0.9)	3 (1.2)		
Vomit				
No	222 (99.6)	242 (100)	1.088	.297
Yes	1 (0.4)	0 (0)		

Abbreviation: HRV, human rhinovirus.

### 3.5 Clinical characteristics of virus infections

We nest analyzed the clinical characteristics of the patients we tested, and found that there was a higher rate of fever (*χ* ^2^ = 11.271, *p* = .001), cough (*χ* ^2^ = 6.987, *p* = .008), runny nose (*χ* ^2^ = 7.980, *p* = .005), and sneeze (*χ* ^2^ = 4.676, *p* = .031) in the positive ones of the infected (Table 5).

**TABLE 5:** The clinical characteristics of the HRV‐tested patients in the outbreaks.

	Negative	Positive	*χ* ^2^	*P*
Age				
Average	11.03	13.4		
Sex				
Male	91 (67.9)	53 (59.6)	1.634	.201
Female	43 (32.1)	36 (40.4)		
Fever				
No	63 (47.0)	22 (24.7)	**11.271**	**.001**
Yes	71 (53.0)	67 (75.3)		
Cough				
No	120 (89.6)	68 (76.4)	**6.987**	**.008**
Yes	14 (10.4)	21 (23.6)		
Sore throat				
No	105 (78.4)	60 (67.4)	3.328	.068
Yes	29 (21.6)	29 (32.6)		
Muscle pain				
No	133 (99.3)	89 (100)	0.667	.414
Yes	1 (0.7)	0 (0)		
Headache				
No	125 (93.3)	80 (89.9)	0.831	.362
Yes	9 (6.7)	9 (10.1)		
Conjunctival congestion				
No	134 (100)	89 (100)	–	–
Yes	0 (0)	0 (0)		
Runny nose				
No	106 (79.1)	55 (61.8)	**7.980**	**.005**
Yes	28 (21.9)	34 (32.8)		
Sneeze				
No	126 (94.0)	76 (85.4)	**4.676**	**.031**
Yes	8 (6.0)	13 (14.6)		
Diarrhea				
No	133 (99.3)	88 (98.9)	0.086	.770
Yes	1 (0.7)	1 (1.1)		
Vomit				
No	133 (99.3)	89 (100)	0.667	.414
Yes	1 (0.7)	0 (0)		

Abbreviation: HRV, human rhinovirus.

## 4 DISCUSSION

The “common cold” was not generally concerned by the general public for the mild symptoms; however, there were more than 250 virus serotypes that could lead to the “common cold” symptoms, and HRV has long been known as the main pathogens of the “common cold,”^2^, ^9^ Cui et al.^10^ have reported that among the influenza‐like illness, 6.46% of which were tested positive for HRV, another study conducted by Zhao et al.^11^ reported that 11.28% of the acute respiratory viral infection were caused by HRV infection. Furuse et al.^12^ also found that rhinoviruses were the most frequently detected pathogen (22.2%) among the acute respiratory infection cases, the hospitalized population would have a higher positive rate of HRV.^13^

A study conducted by Dr. Luka et al.^14^ in Kenya reported that the common cold caused by HRV was mainly detected among school‐going children or teenagers with mild acute respiratory symptoms. However, the HRV‐infected adults had relatively higher mortality and longer hospitalization than the patients infected with IFVs in the elderly population.^15^ Dr. Park et al.^16^ analyzed the etiological surveillance data of respiratory viruses in South Korea from 2016 to 2019 and 2020 and found that the enveloped respiratory viruses (human coronavirus; non‐SARS‐COV‐2; HMPV; IFV; PIV; and RSV) were effectively controlled at the COVID‐19 control measures in 2020, while non‐enveloped respiratory viruses (ADV; HRV; and human bocavirus) were not effectively controlled during this period, and among hospitalized cases, the proportion of hospitalizations due to rhinovirus infection was significantly increased. A study conducted by Dr. Zhang et al.^17^ observed a sharp increase in the positive rate of HRV in Beijing, China, and reasonably assumed to be explained by the reopening of the primary and secondary schools in Beijing. Studies conducted in Shanghai, China, America, and German showed that HRV stayed low in the first quarter of 2020, and raised rapidly in June, matches our study, and observed relatively higher morbidity in the rest of the year.^18^, ^19^, ^20^, ^21^ Another study^22^ conducted in Southampton, UK, gave a theory that the HRV was mainly found in children and adolescents who were the major reservoir for HRV infection, social distancing and face masks are not effective in preventing transmission of HRV, with “*Back‐to‐School Upper Respiratory Infection*” effect,^23^ the risk of HRV transmission surges after schools reopening. On the other hand, Dr. Leung et al.^24^ conducted experiments and proved that there was no significant difference in filtering or protection effect of HRV between wearing a face mask or not, indicating that wearing masks cannot effectively protect the population from the HRV infection.

Our previous studies^25^, ^26^, ^27^ have shown that when combated with the COVID‐19 pandemic, measures had been taken to stop the transmission of the SARS‐COV‐2, and respiratory viruses like the influenza virus, varicella, herpes zoster, rubella, measles, mumps virus were observed all dramatically decreased during the fighting against COVID‐19 pandemic; however, HRV was found remains prevalent in school teenagers.^28^

In our study, most of the outbreaks were reported the primary and secondary schools of Guangzhou, which was agreed with Dr. Luka et al.^14^ and Dr. Zhang et al. study.^17^ Seventy‐four percent of the patients in our study were recorded to have a fever, 30% of the patients had a sore throat and runny nose, 19% of the patients had a cough, and 8% of the patients had sneezing, so the most typical symptom of HRV infection in our study was fever. By sampling and testing, we found that patients with the symptoms of fever, cough, runny nose, and sneezing were more likely to test positive for HRV in an HRV‐related outbreak. Studies have proved that HRV infection in youngsters would be symptomatic while in adults often asymptomatic.^29^ However, as we illustrated in Table 4, the tested patients had a higher proportion of fever, which would sure be selection bias, because the strategy during the control of the outbreaks is to identify the pathogens and control the spreading, so we were more likely to take the specimens of the patients with obvious symptoms instead of collecting samples of all patients, to improve the detection success rate.

Diurnal temperature range (DTR) was reported to be one of the risk factors of the common cold, which gives the answer to the trend of the endemic of HRV in spring and winter of the year.^30^ Obesity was another risk factor reported to rise the possibility of HRV infection.^31^ While in our study, we did not collect the DTR, height, and weight data of the patients, which is a shortcoming of this study.

In summary, this study gives a glimpse of the endemic of HRV in Guangzhou, proved better evidence for the prevention and control of respiratory viruses, and indicated that the conventional COVID‐19 control measures, such as wearing face masks or social distancing, are not effective enough in preventing rhinovirus, so new masks that filter nonenveloped viruses, including HRV, should be considered as a possible new approach to preventing HRV infection.

## AUTHOR CONTRIBUTIONS

**Di Wu，Jianyun Lu and Tiantian Wu**: Conceptualization and investigation of the original draft. **Tiantian Wu, Qing Zeng, and Yiyun Chen**: Methodology. **Yanhui Liu**: Data curation. **Di Wu and Jianyun Lu**: Review and editing.

## CONFLICT OF INTEREST

The authors declare no conflict of interest.

## Data Availability

Raw data were generated during the field investigation. Derived data supporting the findings of this study are available from the corresponding author on request.

## References

[1] Jans DA , Ghildyal R . Rhinoviruses. In: Jans DA , ed. Methods in Molecular Biology. Springer; 2015. 10.1007/978-1-4939-1571-2 25383405

[2] Jacobs SE , Lamson DM , St George K , Walsh TJ . Human rhinoviruses. Clin Microbiol Rev. 2013;26:135‐162. 23297263 10.1128/CMR.00077-12 PMC3553670

[3] Smith ME , Wilson PT . Human rhinovirus/enterovirus in pediatric acute respiratory distress syndrome. J Pediatr Intensive Care. 2020;9:81‐86. 32351760 10.1055/s-0039-3400466 PMC7186013

[4] Takashita E , Kawakami C , Momoki T , et al. Increased risk of rhinovirus infection in children during the coronavirus disease‐19 pandemic. Influenza Other Respir Viruses. 2021;15:488‐94. 33715290 10.1111/irv.12854 PMC8189209

[5] Champredon D , Bancej C , Lee L , Buckrell S . Implications of the unexpected persistence of human rhinovirus/enterovirus during the COVID‐19 pandemic in Canada. Influenza Other Respir Viruses. 2021;16:190‐192. 34747155 10.1111/irv.12930 PMC8652650

[6] Kim HM , Lee EJ , Lee NJ , et al. Impact of coronavirus disease 2019 on respiratory surveillance and explanation of high detection rate of human rhinovirus during the pandemic in the Republic of Korea. Influenza Other Respir Viruses. 2021;15:721‐31. 34405546 10.1111/irv.12894 PMC8446939

[7] Wu A , Mihaylova VT , Landry ML , Foxman EF . Interference between rhinovirus and influenza A virus: a clinical data analysis and experimental infection study. Lancet Microbe. 2020;1:e254‐e262. 33103132 10.1016/s2666-5247(20)30114-2 PMC7580833

[8] Piret J , Boivin G . Viral interference between respiratory viruses. Emerg Infect Dis. 2022;28:273‐281. 35075991 10.3201/eid2802.211727 PMC8798701

[9] Lewis‐Rogers N , Seger J , Adler FR . Human rhinovirus diversity and evolution: how strange the change from major to minor. J Virol. 2017;91:e01659‐e01716. 28100614 10.1128/JVI.01659-16 PMC5355621

[10] Cui X , Teng Z , Zhou Y , Zhao X , Zhang X . Prevalence and genotyping characters of human rhinovirus among influenza‐like illness patients in Shanghai in 2018. Int J Virol. 2020;27:279‐282(in Chinese).

[11] Zhao L , Fang X , Wang M , Liu Y , Chen Y , Jiang H . Epidemiological investigation of 452 children with acute respiratory viral infection. Chin J Woman Child Health Res. 2020;31:722‐726 (in Chinese).

[12] Furuse Y , Tamaki R , Suzuki A , et al. Epidemiological and clinical characteristics of children with acute respiratory viral infections in the Philippines: a prospective cohort study. Clin Microbiol Infect. 2021;27:1037. 10.1016/j.cmi.2020.09.017 32950713

[13] Peltola V , Waris M , Osterback R , Susi P , Hyypia T , Ruuskanen O . Clinical effects of rhinovirus infections. J Clin Virol. 2008;43:411‐414. 18835215 10.1016/j.jcv.2008.08.014

[14] Luka MM , Kamau E , Adema I , et al. Molecular epidemiology of human rhinovirus from 1‐year surveillance within a school setting in rural coastal Kenya. Open Forum Infect Dis. 2020;7:ofaa385. 33094115 10.1093/ofid/ofaa385 PMC7568438

[15] Hung IF , Zhang AJ , To KK , et al. Unexpectedly higher morbidity and mortality of hospitalized elderly patients associated with rhinovirus compared with influenza virus respiratory tract infection. Int J Mol Sci. 2017;18:259. 10.3390/ijms18020259 PMC5343795 28134768

[16] Park S , Michelow IC , Choe YJ . Shifting patterns of respiratory virus activity following social distancing measures for COVID‐19 in South Korea. J Infect Dis. 2021;224:1900‐1906. 34009376 10.1093/infdis/jiab231 PMC8135809

[17] Zhang RX , Chen DM , Qian Y , et al. Surges of hospital‐based rhinovirus infection during the 2020 coronavirus disease‐19 (COVID‐19) pandemic in Beijing, China. World J Pediatr. 2021;17:590‐596. 34713393 10.1007/s12519-021-00477-2 PMC8552974

[18] Jia R , Lu L , Li S , et al. Human rhinoviruses prevailed among children in the setting of wearing face masks in Shanghai, 2020. BMC Infect Dis. 2022;22:253. 35287614 10.1186/s12879-022-07225-5 PMC8919361

[19] Maison N , Peck A , Illi S , et al. The rising of old foes: impact of lockdown periods on "non‐SARS‐CoV‐2" viral respiratory and gastrointestinal infections. Infection. 2022;50:519‐524. 35076891 10.1007/s15010-022-01756-4 PMC8787179

[20] Jiang H , Yang T , Yang C , et al. Molecular epidemiology and clinical characterization of human rhinoviruses circulating in Shanghai, 2012‐2020. Arch Virol. 2022;167:1111‐1123. 35303167 10.1007/s00705-022-05405-x PMC8931777

[21] Uhteg K , Amadi A , Forman M , Mostafa HH . Circulation of non‐SARS‐CoV‐2 respiratory pathogens and coinfection with SARS‐CoV‐2 amid the COVID‐19 pandemic. Open Forum Infect Dis. 2022;9:ofab618. 35211632 10.1093/ofid/ofab618 PMC8863080

[22] Poole S , Brendish NJ , Tanner AR , Clark TW . Physical distancing in schools for SARS‐CoV‐2 and the resurgence of rhinovirus. Lancet Resp Med. 2020;8:e92‐e93. 10.1016/S2213-2600(20)30502-6 PMC7581315 33289636

[23] Perry Markovich M , Glatman‐Freedman A , Bromberg M , et al. Back‐to‐school upper respiratory infection in preschool and primary school‐age children in Israel. Pediatr Infect Dis J. 2015;34:476‐481. 25879647 10.1097/INF.0000000000000627

[24] Leung NHL , Chu DKW , Shiu EYC , et al. Respiratory virus shedding in exhaled breath and efficacy of face masks. Nature Med. 2020;26:676‐680. 32371934 10.1038/s41591-020-0843-2 PMC8238571

[25] Wu D , Ma X , Geng H , et al. Reduction in mumps during the fight against the COVID‐19 pandemic. Asia Pac J Public Health. 2021;33:171‐173. 33143462 10.1177/1010539520971171

[26] Wu D , Lu J , Liu Y , Zhang Z , Luo L . Positive effects of COVID‐19 control measures on influenza prevention. Int J Infect Dis. 2020;95:345‐346. 32283283 10.1016/j.ijid.2020.04.009 PMC7151395

[27] Wu D , Liu Q , Wu T , Wang D , Lu J . The impact of COVID‐19 control measures on the morbidity of varicella, herpes zoster, rubella and measles in Guangzhou, China. Immun Inflamm Dis. 2020;8:844‐846. 33026189 10.1002/iid3.352 PMC7654402

[28] Wu D , Lu J , Sun Z , et al. Rhinovirus remains prevalent in school teenagers during fight against COVID‐19 pandemic. Immun Inflamm Dis. 2020;9:76‐79. 33247521 10.1002/iid3.381 PMC7753714

[29] Peltola V , Waris M , Osterback R , Susi P , Ruuskanen O , Hyypia T . Rhinovirus transmission within families with children: incidence of symptomatic and asymptomatic infections. J Infect Dis. 2008;197:382‐389. 18248302 10.1086/525542

[30] Ma Y , Yang S , Yu Z , et al. Effect of diurnal temperature range on outpatient visits for common cold in Shanghai, China. Environ Sci Pollut Res Int. 2020;27:1436‐1448. 31748999 10.1007/s11356-019-06805-4

[31] Orozco‐Hernandez JP , Montoya‐Martinez JJ , Pacheco‐Gallego MC , Cespedes‐Roncancio M , Porras‐Hurtado GL . SARS‐CoV‐2 and rhinovirus/enterovirus co‐infection in a critically ill young adult patient in Colombia. Biomedica. 2020;40:34‐43. 10.7705/biomedica.5516 PMC7676835 33152186

